# The association of oxidative stress biomarkers with type 2 diabetes mellitus: A systematic review and meta‐analysis

**DOI:** 10.1002/hsr2.389

**Published:** 2021-10-01

**Authors:** Sujan Banik, Antara Ghosh

**Affiliations:** ^1^ Department of Pharmacy Noakhali Science and Technology University Noakhali Bangladesh

**Keywords:** antioxidant, malondialdehyde, meta‐analysis, oxidative stress, type 2 diabetes mellitus

## Abstract

**Background and Aims:**

Oxidative stress plays a major role in the development of type 2 diabetes mellitus (T2DM). However, there were controversial outcomes in the literature between the association of oxidative stress biomarkers and T2DM. The purpose of this systematic review and meta‐analysis was to critically examine the association of oxidative stress biomarkers with T2DM.

**Methods:**

We systematically searched different electronic databases including PubMed/Medline, EMBASE, ScienceDirect, Web of Science, and Cochrane Library to find the relevant studies up to May 2021. The pooled standard mean difference (SMD) with a 95% confidence interval (CI) was used to define the variation between the study groups.

**Results:**

A total of 22 case‐control studies with 2853 subjects (1667 diabetic patients and 1186 healthy controls) were found to be eligible for this meta‐analysis. The pooled results of meta‐analysis showed a significant difference in the levels malondialdehyde (SMD [95% CI]: 2.27 [1.62, 2.91]), nitric oxide (SMD [95% CI]: 1.40 [0.00, 2.81]), glutathione (SMD [95% CI]: −1.76 [−2.94, −0.59]), and total antioxidant status (SMD [95% CI]: −1.40 [−2.28, −0.51]) between the patient group and healthy subjects, whereas no significant difference was observed in the superoxide dismutase levels (SMD [95% CI]: −1.20 [−2.55, 0.15]) and glutathione peroxidase levels (SMD [95% CI]: 0.07 [−2.80, 2.94]).

**Conclusion:**

The present analysis suggests that oxidative stress might have a potential role in the pathogenesis of T2DM in humans. However, further studies should be needed to elucidate the possible mechanism and strengthen this evidence.

## 1 INTRODUCTION

Diabetes mellitus (DM) is a group of metabolic disorders characterized by the elevated levels of glucose in the blood and insufficient secretion or action of endogenous insulin.^1^ A report by the International Diabetes Federation in 2017 showed the worldwide prevalence of diabetes in the adult population reached 8.8% (424.9 million people).^2^ Among them, the majority (87%‐91%) of the cases are with type 2 diabetes. Globally, type 2 diabetes mellitus (T2DM) is considered a major public health concern because of its life‐threatening complications with the increasing risk of mortality.^3^ Although the exact etiologies of T2DM are not well defined, it is believed that autoimmune disease, genetic, and environmental factors play a major role in developing T2DM.^4^ Also, recent studies have shown that with the high level of free‐radical generation, oxidative stress (OS) initiates a significant role in developing and progressing T2DM.^5^, ^6^, ^7^, ^8^

OS can be defined as an imbalance between the production of reactive oxygen species (ROS) and antioxidant defense by which the body can detoxify its harmful effects and inhibit cell damages. The generation of ROS was thought to be a form of pathological cellular stress, but the current investigation is that ROS formed due to the physiological and homeostatic functions of many cells. However, an excess formation of ROS such as superoxide, hydrogen peroxide, and hydroxyl radicals can cause harmful effects on many cellular structures such as protein, lipids, and nucleic acids.^9^ Antioxidants such as catalase (CAT), superoxide dismutase (SOD), glutathione (GSH), and glutathione peroxidase (GPX) counter the action of ROS by neutralizing their action or by inhibiting their formation.^10^ Thus, a balance might be important between ROS and the levels of the antioxidant; otherwise, OS has been implicated in the pathogenesis of a variety of diseases, including cancer, obesity, diabetes mellitus, cardiovascular diseases, and nonalcoholic fatty liver disease.^11^, ^12^ Various studies have reported that significant and abnormal increases in the levels of OS biomarkers associated with T2DM.^13^, ^14^, ^15^, ^16^, ^17^, ^18^, ^19^, ^20^, ^21^ Moreover, Lipiski et al^22^ reported that the decreased levels of enzymatic antioxidants in T2DM leading to the development of diabetic complications. However, the results are controversial.

Therefore, the purpose of this study was to systematically review the evidence of published case‐control studies on this topic and performing a meta‐analysis of the results to summarize and delineate the association between OS and T2DM.

## 2 METHODS

### 2.1 Literature search strategy

We performed this systematic review on OS in diabetes mellitus according to the Preferred Reporting Items for Systematic Reviews and Meta‐Analyses (PRISMA) guidelines^23^ (Data S1). To find relevant peer‐reviewed studies regarding the levels of OS markers, antioxidants status in diabetes mellitus, different electronic databases including PubMed/Medline, EMBASE, ScienceDirect, Web of Science, and Cochrane Library were used. The search terms included “type 2 diabetes mellitus,” “T2DM,” “MDA,” “malondialdehyde,” “oxidative stress,” “antioxidants,” “total antioxidant capacity,” “total antioxidant status,” “superoxide dismutase,” “glutathione,” “glutathione peroxidase,” “nitric oxide,” “catalase,” “vitamin A,” “vitamin C,” and “vitamin E.” The combinations of different search terms were used for identifying the relevant articles, and the search strategies were customized to suit each database. For the present study, ethical approval is not required, as it is a meta‐analysis.

### 2.2 Criteria for inclusion and exclusion

Inclusion criteria for this study were the following: (a) the study must be a case‐control study design; (b) it should be published in a peer‐reviewed journal in the English language; (c) the assessment of OS biomarkers and antioxidant status should be available in both patients and control subjects; (d) studies should have clear diagnostic criteria; and (e) reported studies should be available in full text (not editorial, commentary, or abstract for conferences). Studies were excluded if (a) they were published in other languages than English and contained only qualitative data; (b) there were no healthy control subjects; (c) the subjects have any history of other diseases.

### 2.3 Data extraction and management

Both authors independently performed data extraction using standard extraction spreadsheets from the selected articles based on the inclusion criteria and enlisted in a table. The following items were extracted from each study: author's name (first author), year of publication, country, groups, gender distribution, mean age (years), number of participants (male vs female), MDA concentration, SOD concentration, GSH concentration, GPX concentration, CAT concentration, TAS concentration, and NO concentration. After the extraction of the data, the authors cross‐checked the data tables and resolved any conflicts and inconsistencies during the data extraction process through discussion with each other.

### 2.4 Quality assessment

The quality assessment of all included studies was conducted by using a modified Newcastle‐Ottawa Quality Assessment scale, and we adopted three main criteria for selecting the studies in this systematic review: (a) an appropriate and clear study objective/research question/aim; (b) a detailed description of the study population with a valid methodology; and (c) applicability of results.

### 2.5 Statistical analysis

A statistical software named Review Manager V5.3 (Cochrane Collaboration, Copenhagen, Denmark) was used for the meta‐analysis. We calculated the standard mean difference (SMD) with a corresponding 95% confidence interval (CI) for each parameter using the random‐effects model. The SMD was calculated as the ratio of the mean difference to the pooled SD by the z‐test. The existence of heterogeneity among studies was evaluated using *I* ^*2*^ and its resultant *P*‐value using chi‐squared tests. *I* ^*2*^ values of 25%, 50%, and 75% define the heterogeneity as low, medium, and high heterogeneity, respectively. The random‐effect model and forest plots were adopted as the pooling method, and funnel plots were used to investigate the publication bias. A *P*‐value <.05 was considered a statistically significant difference between groups.

## 3 RESULTS

### 3.1 Search results

**FIGURE 1:**
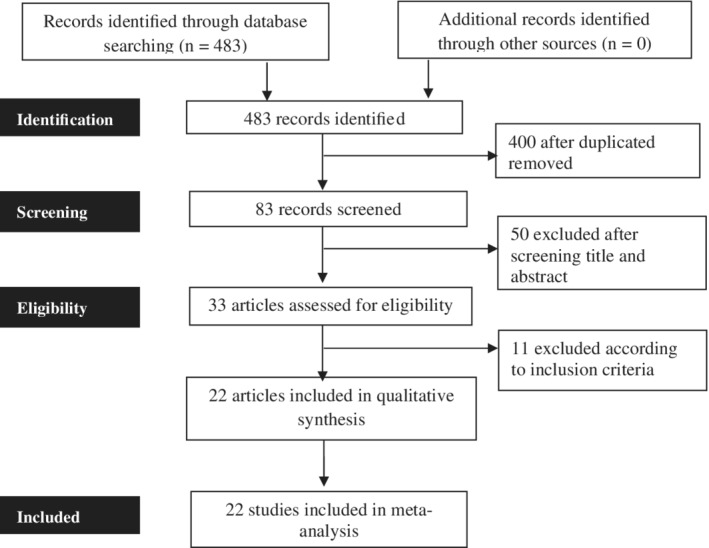
The flow diagram of the literature search and study selection according to the PRISMA guidelines

As shown in Figure 1, a total of 483 studies initially were identified through different database searching. After the removal of duplication, additional screening, and analysis of the titles and abstracts, 33 articles were included as eligible for this study. Of 33 articles, finally, 22 studies were included in the qualitative and quantitative review and meta‐analysis in the study.

### 3.2 Characteristics of included studies

The main characteristics of the included studies published between 1993 and 2018 with 2853 participants (1667 type 2 diabetic patients and 1186 healthy controls) are summarized in Table 1. The studies were conducted in India (n = 9), Turkey (n = 4), China (n = 2), Egypt (n = 2), France (n = 1), Sweden (n = 1), Thailand (n = 1), UAE (n = 1), and Bangladesh (n = 1). All studies included both men and women, except for four studies, in which there was no information about the sex of participants.^17^, ^24^, ^25^, ^26^ The mean value of body mass index (BMI) was higher among the patients compared to the healthy control subjects. Studies evaluated different biomarkers to assess the OS in the patient group compared to control subjects. Among the 22 studies, based on different types of OS biomarkers (MDA/TAS/GPX/GSH/NO/SOD), studies were categorized into the following six groups:

1. A total of 21 studies^13^, ^14^, ^15^, ^16^, ^17^, ^18^, ^19^, ^20^, ^21^, ^24^, ^25^, ^26^, ^27^, ^28^, ^29^, ^30^, ^31^, ^32^, ^33^, ^34^, ^35^, ^36^ reported the association between MDA levels and T2DM (1362 cases and 1168 controls) (Table 2),
2. A total of eight studies^17^, ^24^, ^25^, ^26^, ^30^, ^31^, ^32^, ^33^ reported the association between SOD levels and T2DM (493 cases and 450 controls) (Table 2),
3. A total of six studies^13^, ^17^, ^24^, ^30^, ^31^, ^32^ reported the association between GSH levels and T2DM (382 cases and 327 controls) (Table 2),
4. A total of four studies^24^, ^25^, ^26^, ^30^ reported the association between GPX levels and T2DM (306 cases and 288 controls) (Table 2),
5. A total of five studies^19^, ^20^, ^29^, ^33^ reported the association between TAS levels and T2DM (376 cases and 359 controls) (Table 2), and
6. A total of four studies^20^, ^21^, ^31^, ^36^ reported the association between NO levels and T2DM (217 cases and 176 controls) (Table 2).

**TABLE 1:** Characteristics of the included studies

Study, year	Country	Patient group					Control group					Evaluated parameters
		Number	Male	Female	Age (years)	BMI (kg/m^2^)	Number	Male	Female	Age (years)	BMI (kg/m^2^)	
Gallou et al, 1993	France	60	40	20	55	NA	53	24	29	35	NA	Plasma‐MDA
Sundaram et al, 1996	India	200	NA	NA	49	23.5	180	NA	NA	50	26.0	Plasma‐MDA, SOD, GPX, GSH
Vessby et al, 2002	Sweden	38	14	24	32	23.5	41	22	19	30	23.1	Plasma‐MDA
Bhatia et al, 2003	India	30	12	18	46.9	24.4	30	11	19	46.3	24.3	Serum‐MDA, NO Erythrocyte‐SOD, GSH
Susleyici et al, 2003	Turkey	107	52	55	57.8	22.8	99	46	53	55.5	22.5	Plasma‐MDA, TAS
Memisogullari et al, 2003	Turkey	38	21	17	53.1	NA	18	10	8	49.3	NA	Erythrocyte‐SOD, GSH, GPX
Atli et al, 2004	Turkey	19	9	10	70	NA	15	6	9	72.2	NA	Plasma‐MDA Erythrocyte‐SOD, GSH
Mahboob et al, 2005	India	70	44	26	53	NA	59	33	26	51.5	NA	Serum‐MDA, GSH
Gupta et al, 2006	India	40	NA	NA	40	26.0	50	NA	NA	46	23.0	Serum‐MDA, SOD, GPX
Song et al, 2007	China	113	64	51	52.4	24.7	92	48	44	50.1	24.4	Plasma‐MDA, TAS Erythrocyte‐SOD, GSH
Kamal et al, 2009	Egypt	50	29	21	NA	NA	15	10	5	NA	NA	Serum‐MDA
Tangvarasittichai et al, 2009	Thailand	50	12	38	68.9	25.4	40	14	26	65.5	23.5	Serum‐MDA
Salem et al, 2010	Egypt	50	27	23	NA	NA	15	8	7	NA	NA	Serum‐MDA
Mallick et al, 2011	India	50	27	23	50	23.6	30	16	14	52	24.2	Serum‐MDA
Khemka et al, 2014	India	102	54	48	51.5	22.7	95	49	46	53.1	23.2	Serum‐MDA
Kumari et al, 2014	India	50	25	25	48	NA	50	25	25	48	NA	Serum‐MDA
Rani et al, 2014	India	93	48	45	NA	NA	93	48	45	NA	NA	Serum‐MDA, TAS
Shang et al, 2015	China	28	NA	NA	29	28.5	40	NA	NA	29	27.6	Plasma‐MDA, LPO, SOD, GPX, TAS
Al‐Rawi et al, 2015	UAE	25	NA	NA	50	NA	25	NA	NA	54	NA	Serum‐MDA, SOD, GSH
Mishra et al, 2017	India	92	59	23	52	NA	51	20	31	47	NA	Plasma‐MDA, NO
Kulaksizoglu et al, 2016	Turkey	35	20	15	65.74	NA	35	21	14	67.6	NA	Serum‐MDA, NO, TAS
Banik et al, 2018	Bangladesh	60	34	26	42.96	23.6	60	27	33	45.74	25.1	Serum‐MDA, NO

*Note*: Age and BMI represent mean.

Abbreviations: BMI, body mass index; CAT, catalase; GPX, glutathione peroxidase; GSH, glutathione; MDA, malondialdehyde; NA, not available; NO, nitric oxide; SOD, superoxide dismutase; TAS, total antioxidant status.

**TABLE 2:** Comparison of the level of MDA, SOD, GSH, GPX, CAT, TAS, and NO in the patient and control groups

Study, year	Patient group			Control group		
	Mean	SD	Number	Mean	SD	Number
MDA (mmol/L)						
Gallou et al, 1993	3.11	0.43	60	2.84	0.28	53
Sundaram et al, 1996	3.12	0.30	200	1.87	0.30	180
Vessby et al, 2002	0.49	0.12	38	0.49	0.18	41
Bhatia et al, 2003	4.1	0.11	30	2.89	0.25	30
Susleyici et al, 2003	0.45	0.21	107	0.36	0.15	99
Atli et al, 2004	0.33	0.7	19	0.31	0.06	15
Mahboob et al, 2005	0.26	0.03	70	0.09	0.01	59
Gupta et al, 2006	1.72	0.27	40	0.92	0.24	50
Song et al, 2007	19.13	7.71	113	10.77	5.59	92
Kamal et al, 2009	10.50	3.45	50	5.81	2.38	15
Tangvarasittichai et al, 2009	2.75	0.15	50	1.65	0.12	40
Salem et al, 2010	10.58	3.81	50	5.81	2.39	15
Mallick et al, 2011	7.83	3.26	50	3.70	1.19	30
Khemka et al, 2014	3.21	1.84	102	2.05	0.99	95
Kumari et al, 2014	4.54	0.78	50	2.31	0.61	50
Rani et al, 2014	3.61	0.63	93	1.93	1.51	93
Shang et al, 2015	6.85	0.71	28	4.75	0.62	40
Al‐Rawi et al, 2015	2.38	0.97	25	1.12	0.35	25
Mishra et al, 2017	2.47	0.53	92	1.43	0.23	51
Kulaksizoglu et al, 2016	9.51	2.82	35	10.75	2.57	35
Banik et al, 2018	5.38	1.64	60	2.63	1.63	60
SOD (U/mg Hb)						
Sundaram et al, 1996	2.6	0.3	200	3.3	0.3	180
Bhatia et al, 2003	0.54	0.09	30	1.04	0.12	30
Memisogullari et al, 2003	2.2	0.6	38	2.5	1.0	18
Atli et al, 2004	28.7	6.4	19	29.5	5.7	15
Gupta et al, 2006	5.35	0.36	40	6.83	0.7	50
Song et al, 2007	36.86	8.16	113	30.54	7.39	92
Shang et al, 2015	72.27	18.81	28	117.06	15.63	40
Al‐Rawi et al, 2015	1.48	0.18	25	1.09	0.18	25
GSH (μmol/L)						
Sundaram et al, 1996	48.1	7.5	200	54.0	3.1	180
Bhatia et al, 2003	2.79	1.34	30	3.11	0.88	30
Memisogullari et al, 2003	7.9	2.8	38	10.3	2.9	18
Atli et al, 2004	7.1	1.7	19	8.8	2.4	15
Mahboob et al, 2005	194.8	11.2	70	272.6	12.0	59
Al‐Rawi et al, 2015	2.15	0.87	25	3.22	0.70	25
GPX (U/mg Hb)						
Sundaram et al, 1996	7.2	0.4	200	5.7	0.4	180
Memisogullari et al, 2003	36.2	7.9	38	45.3	10.4	18
Gupta et al, 2006	13.37	0.33	40	14.64	1.43	50
Shang et al, 2015	7.75	1.29	28	10.84	2.84	40
TAS (μmol/L)						
Susleyici et al, 2003	1.43	0.21	107	1.40	0.13	99
Song et al, 2007	11.07	4.42	113	15.08	3.31	92
Rani et al, 2014	0.46	0.46	93	1.69	1.34	93
Shang et al, 2015	6.30	1.00	28	10.56	1.82	40
Kulaksizoglu et al, 2016	1.15	0.16	35	1.48	0.11	35
NO (μmol/L)						
Bhatia et al, 2003	50.2	36.2	30	36.7	7.40	30
Mishra et al, 2017	12.76	1.43	92	7.44	1.26	51
Kulaksizoglu et al, 2016	19.43	8.75	35	13.89	7.71	35
Banik et al, 2018	47.20	70.88	60	15.86	14.95	60

Abbreviations: GPX, glutathione peroxidase; GSH, glutathione; MDA, Malondialdehyde; NO, nitric oxide; SOD, superoxide dismutase; TAS, total antioxidant status.

### 3.3 Association between MDA and T2DM

**FIGURE 2:**
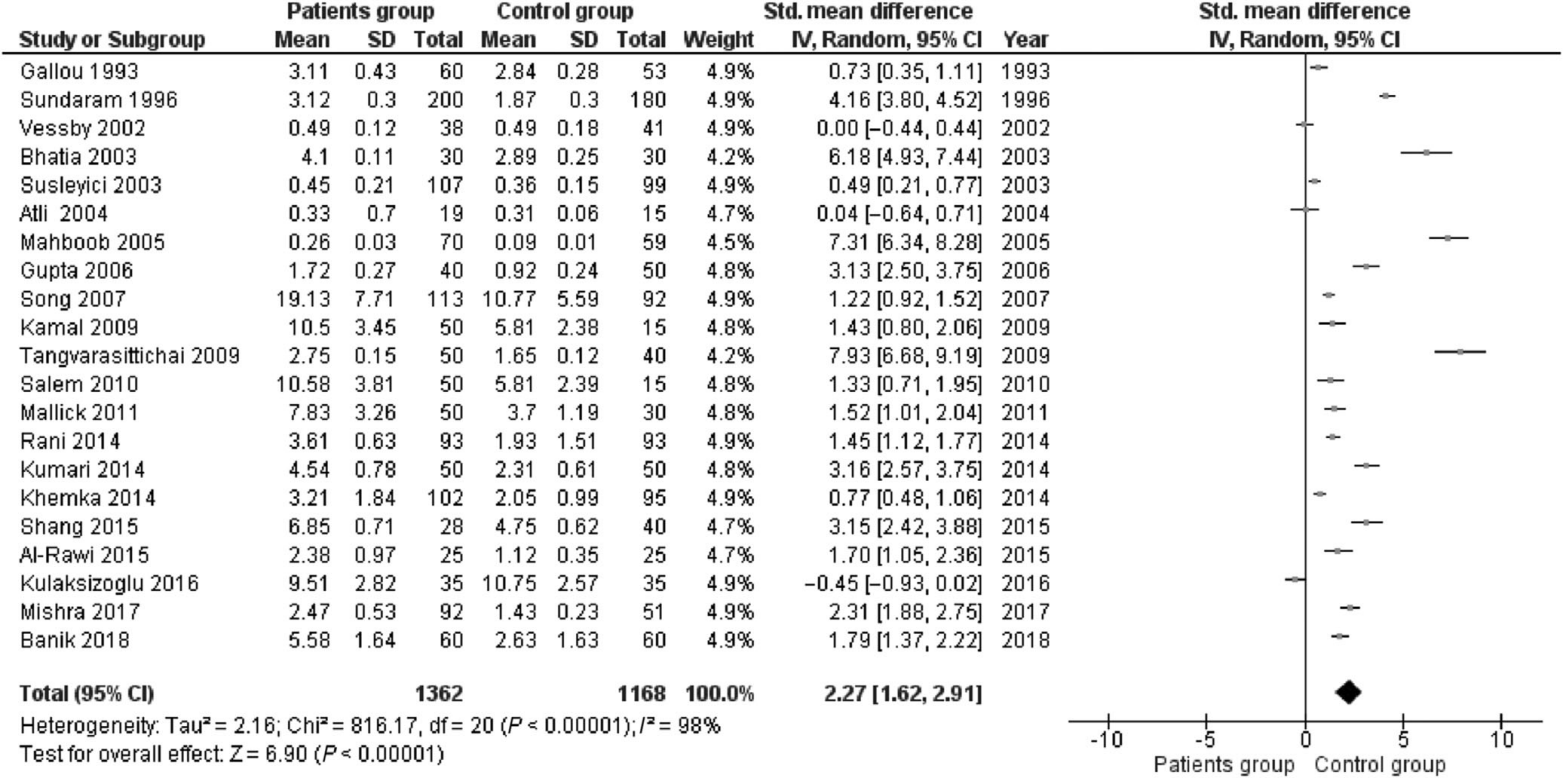
Forest plot of the random effects in a meta‐analysis, showing the association of malondialdehyde with diabetes. The square denotes an effect estimate of individual studies with 95% confidence interval (CI) with the size of squares related to the weight assigned to the study in the meta‐analysis

There were 21 studies to be included in the meta‐analysis to evaluate the overall effect of MDA in T2DM. Based on the random‐effects model of meta‐analysis, significantly higher levels of serum MDA were observed in the patient group compared to the control subjects (SMD [95% CI]: 2.27 [1.62, 2.91], z = 6.90, *P* < .00001). We found a significant level of heterogeneity for MDA among the existing studies (*I* ^*2*^ = 98%, *P* < .00001) (Figure 2).

### 3.4 Association between SOD and T2DM

**FIGURE 3:**
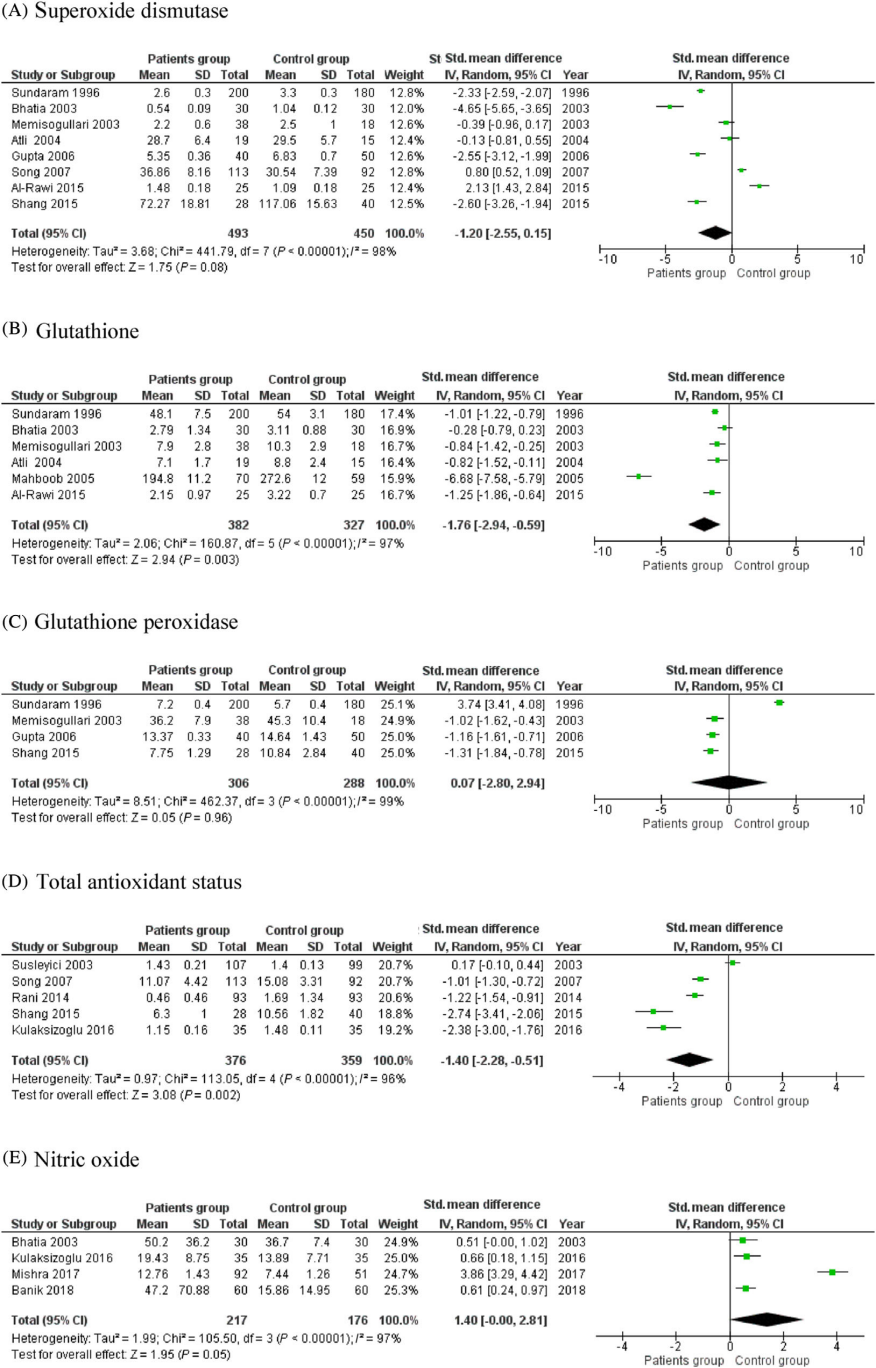
Forest plot of the random effects in a meta‐analysis, showing the association of (A) superoxide dismutase, (B) glutathione, (C) glutathione peroxidase, (D) total antioxidant status, and (E) nitric oxide with diabetes. The square denotes an effect estimate of individual studies with 95% confidence interval (CI) with the size of squares related to the weight assigned to the study in the meta‐analysis

The meta‐analysis of eight included studies in this systematic review revealed a lower level of SOD in patients with T2DM compared to the controls, but the difference was not statistically significant (SMD [95% CI]: −1.20 [−2.55, 0.15], z = 1.75, *P* = .08). On the other hand, we observed a significant level of heterogeneity among the included studies for SOD (*I* ^*2*^ = 98%, *P* < .00001) (Figure 3A).

### 3.5 Association between GSH and T2DM

Random‐effects modeling of the meta‐analysis revealed significantly lower levels of GSH in patient group compared to the control subjects (SMD [95% CI]: −1.76 [−2.94, −0.59], z = 2.94, *P* = .003] with significant heterogeneity (*I* ^*2*^ = 97%, *P* < .00001) (Figure 3B).

### 3.6 Association between GPX and T2DM

We found four papers that reported the GPX activity in diabetic patients. The meta‐analysis revealed that there was no significant difference observed in the level of GPX in the patient group when compared to the control group (SMD [95% CI]: 0.07 [−2.80, 2.94], z = 0.05, *P* = .96) (Figure 3C).

### 3.7 Association between TAS and T2DM

The meta‐analysis of five included studies reported on TAS activity revealed that the TAS level was significantly lowered in the patient group compared to the control group (SMD [95% CI]: −1.40 [−2.28, −0.51], z = 3.08, *P* = .002) with the significant level of heterogeneity (*I* ^*2*^ = 96%, *P* < .00001) (Figure 3D).

### 3.8 Association between NO and T2DM

Based on a random‐effects meta‐analysis, comparing the NO level between the patient group and the control group, a significant difference was obtained for NO levels with a higher level in patients (SMD [95% CI]: 1.40 [0.00, 2.81], z = 1.95, *P* = .05). We observed a significant level of heterogeneity for NO level among the existing studies (*I* ^*2*^ = 97%, *P* < .00001) (Figure 3E).

### 3.9 Publication bias

A funnel plot was used to analyze the publication bias in this systematic review and meta‐analysis. The visual inspection of funnel plots of OS biomarkers in DM did not suggest potential publication bias except for GPX (Data S2).

## 4 DISCUSSION

Many studies have reported that OS is involved in the pathogenesis of multiple disorders including type 1 and type 2 diabetes.^1^, ^4^ To our best knowledge, this is the first systematic review and meta‐analysis to find out the evidence on the association of OS biomarkers such as MDA, SOD, GSH, GPX, TAS, and NO levels in patients with T2DM. From our meta‐analysis, we found significantly higher levels of MDA and NO (considered as oxidants), and significantly lower levels of GSH and TAS (known as antioxidants) in patients compared to control subjects. On the other hand, we observed there was no significant difference in the levels of SOD and GPX between patients and control subjects. The overall results revealed that the impaired oxidants and antioxidants balance play a vital role in the pathogenesis of T2DM.

In this meta‐analysis, we observed a significantly increased level of MDA in patients with T2DM in almost all studies. Both experimental and clinical studies revealed that free radicals are formed in T2DM by glucose degradation, nonenzymatic glycation of proteins, and subsequent oxidative degradation.^37^, ^38^, ^39^ The increased levels of free radicals may lead to lipid peroxidation and the level of MDA is usually measured as a well‐known marker of lipid peroxidation.^40^, ^41^ As a marker of oxidants, the level of NO in diabetic patients was reported in this study, and the analysis revealed a higher level in patients compared to that of control groups.

It has been reported that the increase in lipid peroxidation is strongly associated with a decline in enzymatic and nonenzymatic antioxidant defense mechanisms.^42^ This meta‐analysis revealed that the levels of TAS and GSH were significantly lower in diabetic patients compared with control subjects. However, there was no association found in SOD and GPX with T2DM in this study. The included studies regarding the association of SOD and GPX levels with T2DM are limited and further studies with a larger sample size should be conducted to confirm the true association. The consequences of this imbalance between oxidants and antioxidants, that is, OS in T2DM can promote the development of complications in patients. A previous study reported that a decreased level of GSH can contribute to β‐cell dysfunction and be involved in the pathogenesis of long‐term complications of diabetes.^43^

Some studies advocated that the dietary supplementation of antioxidants like GSH precursor amino acid and antidiabetic drugs like gliclazide and metformin helps scavenge the free radicals and reduces oxidative damage in the face of persistent hyperglycemia.^36^, ^44^, ^45^, ^46^ The role of gliclazide and metformin to increase the antioxidant capacity of erythrocyte CAT, GPX, and glutathione S‐transferase enzyme in treated patients and reduced the OS in diabetes. The polyphenol‐rich fruit has a significant effect on GSH levels owing to their antioxidant activity and also reported that polyphenol‐containing natural fruits play important role in scavenging free radicals and ROS, resulting in protect the cellular damage from free radicals.

Although our study followed a standard search strategy in the current meta‐analysis, this study has some limitations. The major limitation of this study is that firstly, significant heterogeneity was encountered perhaps due to various regimens, doses, duration, study settings, population enrolled, etc for the analysis of OS biomarkers in the included studies. Secondly, we did not analyze any correlation between the complications of T2DM with OS biomarkers due to inadequate data and also sensitivity analysis of the included studies. Finally, subgroup analysis and meta‐regression could not be performed in this meta‐analysis due to the limited studies in the literature.

## 5 CONCLUSION

This systematic review and meta‐analysis provide evidence that the increased OS has a major role in the pathogenesis and progression of T2DM. Therefore, further studies are needed to strengthen this evidence, especially on the association of SOD and GPX levels with T2DM.

## FUNDING

This study did not receive any specific grant from any organizations like the public or commercial.

## CONFLICT OF INTEREST

We have declared that we have no competing interests.

## AUTHOR CONTRIBUTIONS

Conceptualization: Sujan Banik.

Formal Analysis: Sujan Banik, Antara Ghosh.

Methodology: Antara Ghosh.

Supervision: Sujan Banik.

Writing–Original Draft Preparation: Sujan Banik.

Writing–Review and Editing: Sujan Banik, Antara Ghosh.

All authors have read and approved the final version of the manuscript.

Sujan Banik had full access to all of the data in this study and takes complete responsibility for the integrity of the data and the accuracy of the data analysis.

## TRANSPARENCY STATEMENT

Sujan Banik affirms that this manuscript is an honest, accurate, and transparent account of the study being reported, that no important aspects of the study have been omitted, and that any discrepancies from the study as planned (and, if relevant, registered) have been explained.

## Supporting information

**Data S1.** PRISMA‐2020 Guideline's checklist

**Data S2.** Funnel plot analysis to detect the publication bias, (a) malondialdehyde, (b) superoxide dismutase, (c) glutathione, (d) glutathione peroxidase, (e) total antioxidant status, and (f) nitric oxide levels in patients with diabetes mellitus.
