# Supplementary figures and images for: The association of oxidative stress biomarkers with type 2 diabetes mellitus: A systematic review and meta‐analysis
Source: Health Sci Rep. 2021 Oct 1;4(4):e389. doi: 10.1002/hsr2.389 (PMC8485598; doi:10.1002/hsr2.389)

**Supplementary file 2 (Fig. 1S):**

(a)

(b)


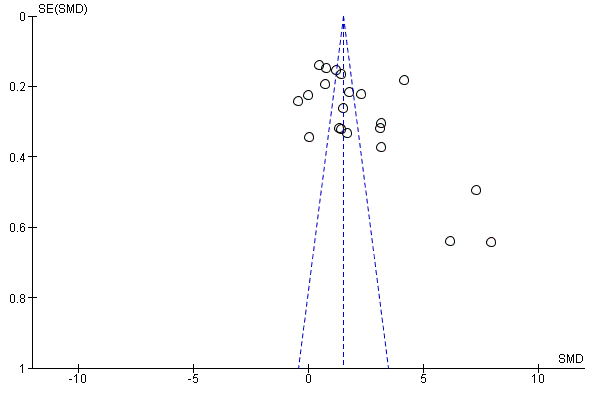

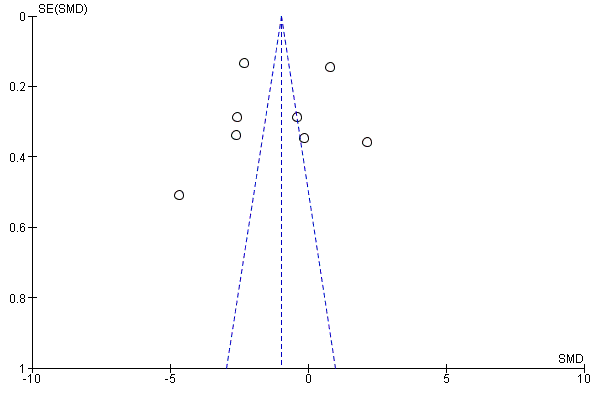


(d)

(c)


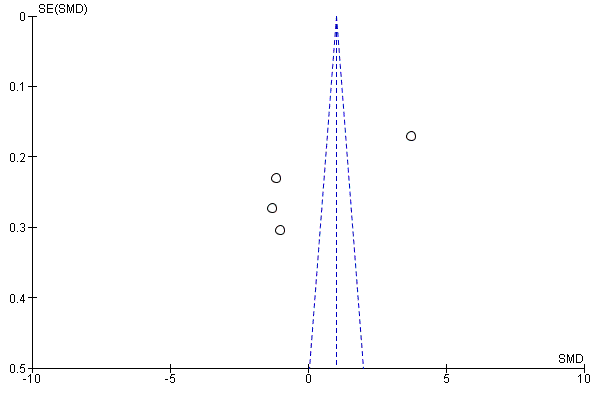

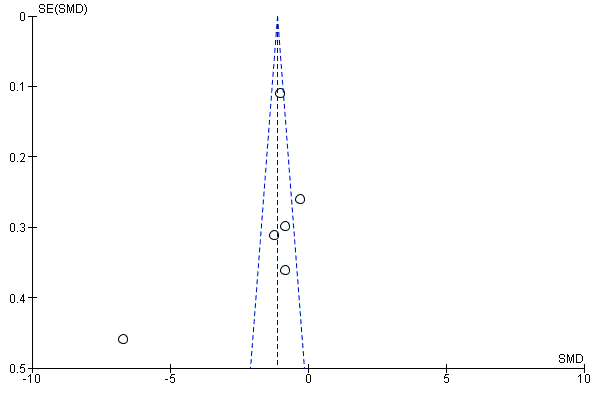


(f)

(e)


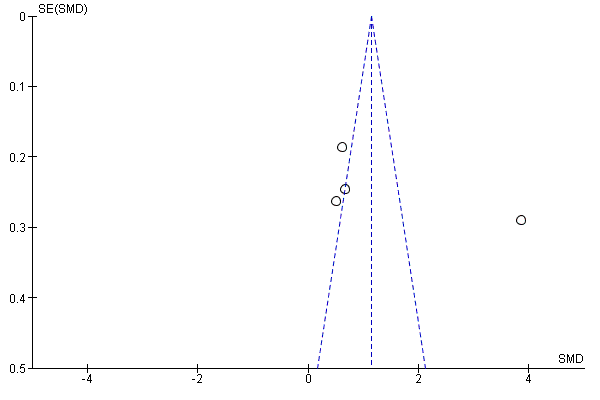


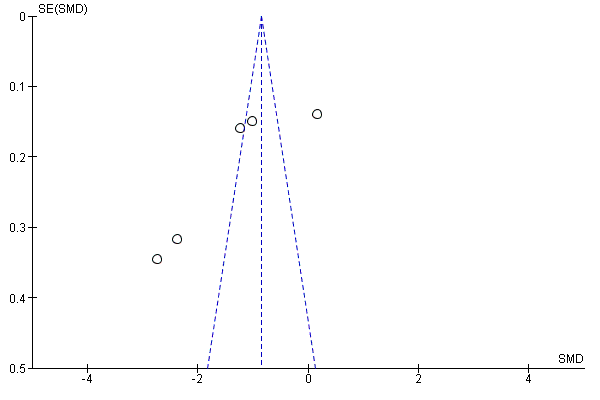

Supplement: Supplementary file 2 — Data S2. Funnel plot analysis to detect the publication bias, (a) malondialdehyde, (b) superoxide dismutase, (c) glutathione, (d) glutathione peroxidase, (e) total antioxidant status, and (f) nitric oxide levels in patients with diabetes mellitus. [file HSR2-4-e389-s001.docx]
